# Gallium-68-Labeled KISS1-54 Peptide for Mapping KISS1 Receptor via PET: Initial Evaluation in Human Tumor Cell Lines and in Tumor-Bearing Mice

**DOI:** 10.3390/ph17010044

**Published:** 2023-12-27

**Authors:** Ina Israel, Gabriele Riehl, Elke Butt, Andreas K. Buck, Samuel Samnick

**Affiliations:** 1 Department of Nuclear Medicine, University Hospital Würzburg, Oberdürrbacher Straße 6, 97080 Würzburg, Germany; israel_i@ukw.de (I.I.); riehl_g@ukw.de (G.R.); buck_a@ukw.de (A.K.B.); 2 Institute of Experimental Biomedicine II, University Hospital Würzburg, Josef-Schneider-Straße 2, 97080 Würzburg, Germany; butt_e@ukw.de

**Keywords:** [^68^Ga]KISS1-54, KISS1-54, KISS1 receptor, GPR54, kisspeptin, human tumor cell lines, positron emission tomography, PET

## Abstract

Kisspeptins (KPs, KISS1) and their receptor (KISS1R) play a pivotal role as metastasis suppressor for many cancers. Low or lost KP expression is associated with higher tumor grade, increased metastatic potential, and poor prognosis. Therefore, KP expression has prognostic relevance and correlates with invasiveness in cancers. Furthermore, KISS1R represents a very promising target for molecular imaging and therapy for KISS1R-expressing tumors. The goal of this study was to evaluate the developed KISS1-54 derivative, [^68^Ga]KISS1-54, as a PET-imaging probe for KISS1R-expressing tumors. The NODAGA-KISS1-54 peptide was labeled by Gallium-68, and the stability of the resulting [^68^Ga]KISS1-54 evaluated in injection solution and human serum, followed by an examination in different KISS1R-expressing tumor cell lines, including HepG2, HeLa, MDA-MB-231, MCF7, LNCap, SK-BR-3, and HCT116. Finally, [^68^Ga]KISS1-54 was tested in LNCap- and MDA-MB-231-bearing mice, using µ-PET, assessing its potential as an imaging probe for PET. [^68^Ga]KISS1-54 was obtained in a 77 ± 7% radiochemical yield and at a >99% purity. The [^68^Ga]KISS1-54 cell uptake amounted to 0.6–4.4% per 100,000 cells. Moreover, the accumulation of [^68^Ga]KISS1-54 was effectively inhibited by nonradioactive KISS1-54. In [^68^Ga]KISS1-54-PET, KISS1R-positive LNCap-tumors were clearly visualized as compared to MDA-MB-231-tumor implant with predominantly intracellular KISS1R expression. Our first results suggest that [^68^Ga]KISS1-54 is a promising candidate for a radiotracer for targeting KISS1R-expressing tumors via PET.

## 1. Introduction

Kisspeptins (KPs) summarize the family of KISS1 peptides, which bind at the KISS1 receptor (KISS1R or GPR54) with high affinity. Among the KPs, KISS1-54 is the best characterized representative (K_i_ 1.45 ± 0.1 nM for KISS1-54) [1,2]. The *Kiss1* gene was first identified as a metastasis suppressor gene in melanoma, which suppresses metastasis without affecting the formation of primary tumor [3]. Since then, Kisspeptin was also identified in pancreatic, lung, breast, ovarian, gastric, colon, bladder, esophageal, and small intestine cancer [4,5,6,7,8,9]. 

The KISS1R belongs to the G protein-coupled receptors activating the G protein G_αq/11_. KISS1R has been found largely in many human cancers, while its expression in most healthy tissues is relatively low [1,2]. The activation of KISS1R by KPs causes extensive downstream signal transduction, such as a decrease in expression of matrix metalloproteinase 9, which reduces the degradation of the extracellular matrix, leading to decreased cell motility and invasion [10]. Moreover, via the Gα_q/11_ cascade after KISS1R activation, the intracellular Ca^2+^ level increases and suppress cell motility [11]. The underlying clinical data for most tumors, e.g., melanoma, ovarian, bladder, renal cell cancer, esophageal squamous cell, and prostate carcinoma, demonstrated that a downregulation of KP/KISS1R correlates with metastasis and a worse prognosis for patients [3,6,12,13,14,15,16]. These findings support the pivotal role of the KP/KISS1R system as a metastasis suppressor. However, further studies convincingly demonstrated a significant increase in the KP/KISS1R expression in primary tumors of higher or invasive grade and in metastatic tumors [17,18]. Particularly in estrogen receptor-alpha (ERα)-negative breast cancer, the KISS1R activation has been reported to increase cell migration and invasiveness, and thus ERα appears to negatively regulate KISS1R-mediated cell invasion [19,20,21]. Thus, contrary to its anti-metastatic role in many tumors, KP/KISS1R might also exhibit pro-metastatic properties, depending on the tumor entity.

Since KISS1R is either lost in higher-grade tumors or specifically expressed in some tumor entities, such as breast cancer, the expression of KISS1R has been considered a very interesting prognostic marker that can provide valuable information on tumor grade and prognosis, depending on the tumor entity [9,12,14,15,21,22]. In addition, the KISS1 receptor represents a promising target for molecular imaging by positron emission tomography (PET), allowing for the noninvasive detection of KISS1R-expressing tumors. On the one hand, addressing KISS1R should open up the possibility of the prognosis of progression in tumor patients. On the other hand, KISS1R-positive tumor patients could be selected for subsequent individualized radionuclide therapy with the corresponding α/β^−^-emitting KISS1R radioligands.

In the present study, we aimed to develop a KISS1-54-based radiotracer for the visualization and quantification of KISS1R in KISS1R-expressing tumors, using PET. Thus, we developed [^68^Ga]NODAGA-KISS1-54 ([^68^Ga]KISS1-54) and tested its stability in injection solution and human serum for further biological evaluations. Furthermore, we investigated the [^68^Ga]KISS1-54 cell uptake in the selected human cell lines of different tumor entities in both in vitro and in vivo in tumor-bearing mice, using µ-PET.

## 2. Results

### 2.1. Radiochemistry

**Figure 1:**
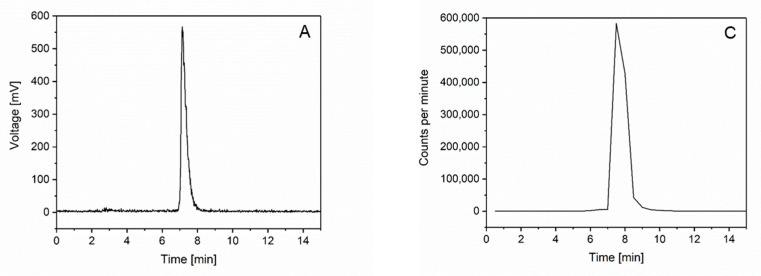

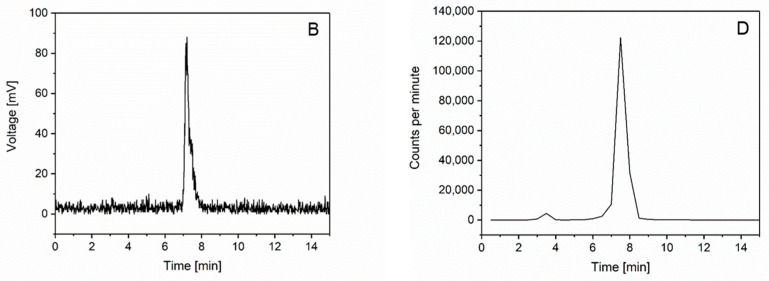
Radiochemical purity and in vitro and in vivo stability. The radiochemical purity of [^68^Ga]KISS1-54 at the EOS was > 99% (**A**) and remained at > 99% for at least 3 h after EOS (**B**), as determined by radio-HPLC. The in vivo stability directly after EOS (**C**) and after 3 h (**D**). Despite the slight decrease to 95.6%, [^68^Ga]KISS1-54 is largely stable in vivo.

Starting from the KISS1R-selective NODAGA-KISS1-54 peptide, we established the synthesis of [^68^Ga]NODAGA-KISS1-54 ([^68^Ga]KISS1-54) in a one-step radiolabeling process. The decay-corrected radiochemical yield of [^68^Ga]KISS1-54 was 77 ± 7%, with a radiochemical purity of >99%, as assessed by radio-HPLC (Figure 1A). The synthesis, including purification, formulation, and subsequent quality control, was completed after 35 min. [^68^Ga]KISS1-54 was stable in the injection solution: 3 h after preparation, the radiochemical purity of the new radiotracer remained >99% (Figure 1B). For the evaluation of the stability in vivo, [^68^Ga]KISS1-54 was incubated in human serum directly at the end of synthesis (EOS). After 1 h, the radiochemical purity decreased to 98.0% (Figure 1C) and declined continuously to 95.6% after 3 h (Figure 1D), confirming the high in vivo stability of [^68^Ga]KISS1-54 over the period of analysis. 

### 2.2. Examination of KISS1R Expression by Immunofluorescence and Western Blot

**Figure 2:**
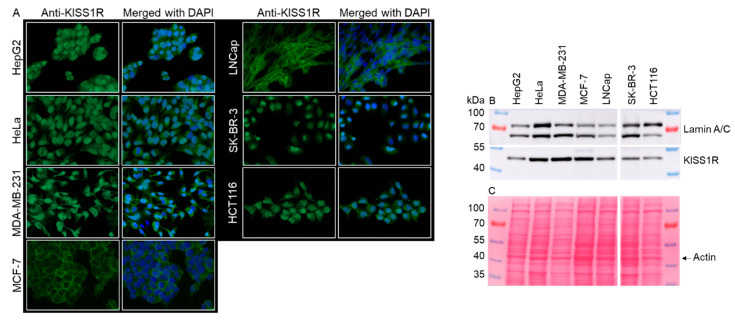
Investigation of KISS1R expression via immunofluorescence and Western blot. (**A**) Immunofluorescence staining against KISS1R and nucleus counterstaining with DAPI in HepG2, HeLa, MDA-MB-231, MCF-7, LNCap, SK-BR-3, and HCT116. The KISS1 receptor expression is proved in all cell lines. (**B**) In addition, KISS1R expression (43 kDa) was confirmed by Western blot in all cell lines examined, but KISS1R appears to be differentially expressed. (**C**) Ponceau-S staining after protein transfer. Comparable protein loading/transfer was detectable; thus, different Lamin A/C intensities can be explained by different expressions in the examined cells.

In order to identify appropriated tumor cell lines expressing KISS1R for in vitro binding studies, we examined different human tumor cell lines of various tumor types both by immunofluorescence (IF) and Western blot (WB). All selected cell lines, HepG2, HeLa, MDA-MB-231, MCF-7, LNCap, SK-BR-3, and HCT116, revealed marked KISS1R expression by both IF and WB (Figure 2A,B). Thus, the investigated target for [^68^Ga]KISS1-54 was present in all cell lines. However, the results from the Western blot clearly determined different amounts of KISS1R protein in the cell lysates obtained from the same number of cells (Figure 2B). The varying lamin bands in the WB can be explained by the different Lamin expressions of the cells, as the Pouceau-S staining showed similar amounts of loaded peptide (Figure 2C).

### 2.3. Investigation of [^68^Ga]KISS1-54 in Human Tumor Cell Lines 

**Figure 3:**
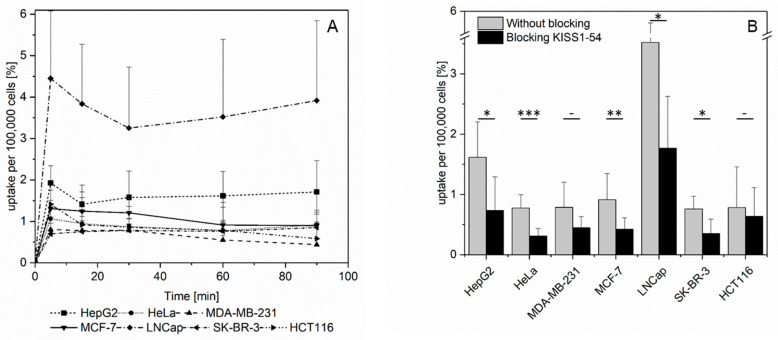
In vitro uptake and blocking studies. (**A**) In vitro cell uptake of [^68^Ga]KISS1-54 in the human cell lines HepG2, HeLa, MCF-7, MDA-MB-231, LNCap, SK-BR-3, and HCT116 over 90 min in% per 100,000 cells. LNCap showed the highest cell uptake of up to 4.4%. For the other cell lines, the cell uptake was significantly lower, ranging from 0.4 to 1.9%. (**B**) Blocking of [^68^Ga]KISS1-54 uptake after 60 min of co-incubation with the nonradioactive KISS1-54 peptide (1,000-fold concentrated) versus the [^68^Ga]KISS1-54 uptake after 60 min without blocking substance. ^—^ *p* > 0.05, * *p* < 0.05, ** *p* < 0.01, and *** *p* < 0.001.

After the KISS1R expression was analyzed, we investigated the [^68^Ga]KISS1-54 accumulation in these cells via cell uptake studies. The results are shown in Figure 3A. The highest uptake levels were virtually reached after 5 min and remained nearly constant over a 90 min incubation time. The highest uptake of [^68^Ga]KISS1-54 was observed in LNCap cells and was 3.3 to 4.4% per 100,000 cells. HepG2 showed the second highest uptake of the radiotracer, ranging from 1.2 to 2.2% per 100,000 cells, followed by MCF-7, with an [^68^Ga]KISS1-54 accumulation between 1.3 and 0.9% per 100,000 cells. The lowest uptake levels were measured in HCT116 (1.4–0.6% per 100,000 cells), HeLa (1.1–0.8% per 100,000 cells), SK-BR-3 (0.9–0.8% per 100,000 cells), and MDA-MB-231 (0.8–0.4% per 100,000 cells). However, after comparing the cellular uptake of [^68^Ga]KISS1-54 of the respective cell lines (Figure 3A) with their expressed KISS1R amount in the Western blot (Figure 2B), no correlation was detected.

In addition, the specificity of the [^68^Ga]KISS1-54 uptake into tumor cells was determined via the co-incubation of [^68^Ga]KISS1-54 with the nonradioactive KISS1-54 peptide in excess for 60 min. In all cell lines, we found a reduced [^68^Ga]KISS1-54 uptake. In detail, compared to the control experiments without KISS1-54 co-incubation that were performed in parallel, the accumulation of [^68^Ga]KISS1-54 was significantly reduced in HepG2 to 46% (*p* = 0.02), in HeLa to 40% (*p* < 0.001), in MCF-7 to 47% (*p* = 0.004), in LNCap to 50% (*p* = 0.047), and in SK-BR-3 to 46% (*p* = 0.02). These results indicate a specific binding of [^68^Ga]KISS1-54 to KISS1R. Only in MDA-MB-231 and HCT116 did the [^68^Ga]KISS1-54 uptake did not significantly decrease to 65% (*p* = 0.08) and 76% (*p* = 0.07), respectively (Figure 3B). 

**Figure 4:**
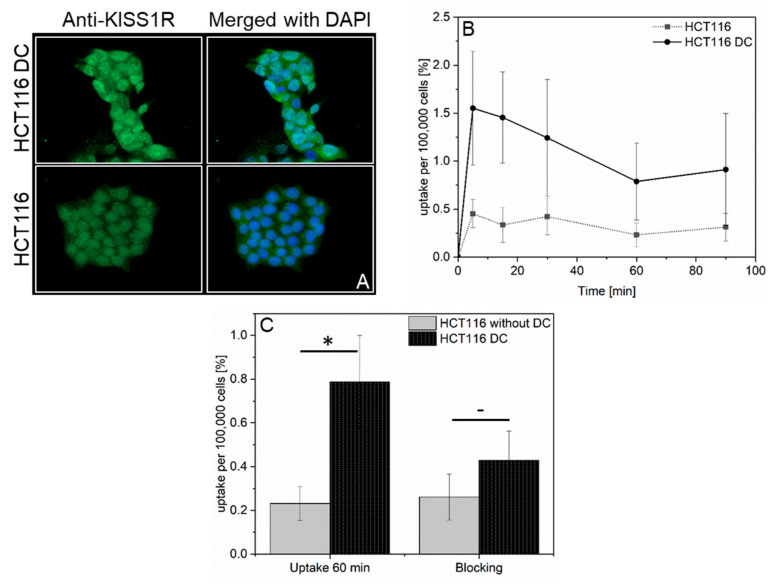
KISS1R expression after pre-incubation with Decitabine. (**A**) Increased KISS1R expression in HCT116 after treatment with Decitabine (DC). Left panel: Anti-KISS1R of HCT116 after treatment with 5 µM DC (top) and without treatment (bottom). Right panels: Merged images of Anti-KISS1R IHC and nuclear staining using DAPI. (**B**) DC treatment increased the [^68^Ga]KISS1-54 uptake in HCT116 (black line) compared to the uptake without DC pre-incubation (dotted line). (**C**) Left side: [^68^Ga]KISS1-54 uptake at 60 min in HCT116 and in HCT116 treated with DC. The [^68^Ga]KISS1-54 uptake in HCT116 could be reduced when [^68^Ga]KISS1-54 was co-incubated with KISS1-54. ^—^ *p* > 0.05, * *p* < 0.05.

Next, we wanted to examine if the pharmacological stimulation of KISS1R expression results in a higher cellular [^68^Ga]KISS1-54 accumulation. In a previous study, Kang et al. showed that the loss of KISS1R expression might be associated with *KISS1R* promotor methylation [23]. Therefore, HCT116 with a low KISS1R level was pre-incubated with 5-Aza-2′-Deoxycytidine (Decitabine), a demethylating cytostatic that inhibits DNA-methyltransferase, resulting in the hypomethylation of gene promotors and reactivation of *KISS1R* gene expressions. The results from IF (Figure 4A) clearly showed that the pre-incubation of HCT116 cells with Decitabine (DC) resulted in an increase in the KISS1R level. Furthermore, the [^68^Ga]KISS1-54 uptake was significantly increased in the DC-pre-incubated cells as compared to that shown in the data obtained for untreated cells (Figure 4B). Moreover, both the DC-treated HCT116 and HCT116 cells without treatment were co-incubated with [^68^Ga]KISS1-54 and KISS1-54. As shown in Figure 4C, the increased [^68^Ga]KISS1-54 uptake resulting from DC treatment could be blocked by co-incubation with KISS1-54 peptide. Therefore, it could be concluded that [^68^Ga]KISS1-54 binds specifically to KISS1R.

### 2.4. In Vivo Studies in Mice

**Figure 5:**
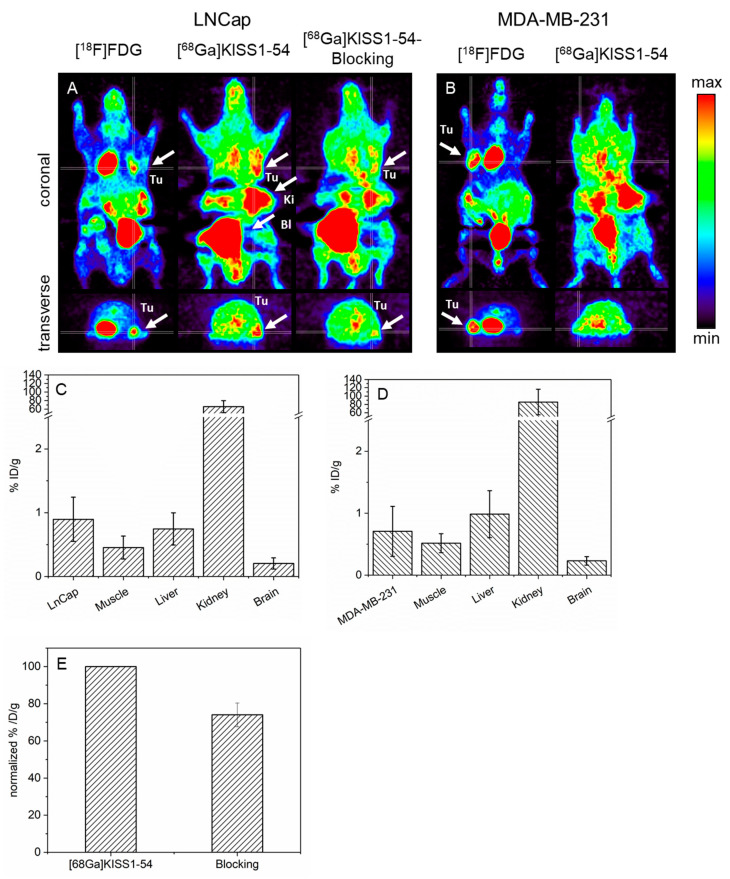
In vivo PET imaging. (**A**) Representative µ-PET images of a CD1 nu/nu mouse with a subcutaneously implanted LNCap tumor on the right anterior flank (white arrow Tu). Left panel: Control [^18^F]FDG-PET to verify tumor vitality and localization. Middle panel: [^68^Ga]KISS1-54-PET of the same LNCap-bearing mouse. The tumor was clearly delineated from the surrounding tissue (white arrow, Tu). In addition, a high [^68^Ga]KISS1-54 accumulation was found in the kidney and bladder, predominantly indicating renal clearance (white arrow Ki and Bl). Right panel: [^68^Ga]KISS1-54-PET after co-injection of [^68^Ga]KISS1-54 with nonradioactive KISS1-54. Visual inspection revealed a marked decrease in [^68^Ga]KISS1-54 uptake. (**B**) Representative µ-PET images of a mouse with a left subcutaneously implanted MDA-MB-231 tumor. Left panel: Clear [^18^F]FDG tumor uptake was demonstrated, indicating that the tumor is vital and well grown (white arrow, Tu). Right panel: [^68^Ga]KISS1-54 PET image of the same mouse. No distinct [^68^Ga]KISS1-54 tumor uptake can be detected. (C/D) Semi-quantification of the [^68^Ga]KISS1-54 in vivo uptake in mice with LNCap (**C**) and MDA-MB-231 (**D**) tumors. (**E**) Quantified blockage [^68^Ga]KISS1-54 tumor uptake in LNCap-bearing mice after co-injection of [^68^Ga]KISS1-54 and KISS1-54 peptide in 100-fold excess.

To confirm the results obtained for the in vitro experiments, [^68^Ga]KISS1-54 was subsequently evaluated in vivo in tumor xenografts in mice. For this purpose, we implanted LNCap, the cell line with the highest [^68^Ga]KISS1-54 in vitro uptake; and MDA-MB-231, the cell line with the lowest in vitro uptake, subcutaneously into the front flank of CD1 nu/nu mice. PET imaging was performed after the intravenous application of [^68^Ga]KISS1-54. Representative µ-PET images of mice bearing an LNCap tumor and an MDA-MB-231 tumor, respectively, are shown in Figure 5A,B. [^68^Ga]KISS1-54 accumulated into the LNCap xenograft (*n* = 8) was clearly delineated from the surrounding tissue (Figure 5A, middle panel). In contrast, there was no significant uptake of [^68^Ga]KISS1-54 in the MDA-MB-231 tumor (*n* = 10) (Figure 5B right panel). Tumor vitality was confirmed with a [^18^F]FDG-PET scan the day before [^68^Ga]KISS1-54 application (Figure 5A,B, left panel). Both the LNCap and MDA-MB-231 tumors showed a clear FDG uptake, confirming the vitality of the implanted tumor xenografts. 

For the quantification, we calculated the percent injected dose per gram (mean) into tumor tissue, muscle, liver, kidney, and brain 60 min p.i. The [^68^Ga]KISS1-54 uptake was 0.9 ± 0.3% ID/g in LNCap and 0.7 ± 0.3% ID/g in MDA-MB-231. The tracer accumulation in the muscle, liver, and brain was about 0.5 ± 0.2% ID/g, 0.8 ± 0.3% ID/g, and 0.2 ± 0.1% ID/g, respectively, and comparable in mice with LNCap and MDA-MB-231. We observed a rapid renal tracer clearance during the first few minutes. At the time point of quantification, the [^68^Ga]KISS1-54 kidney activity in LNCap- and MDA-BA-231-bearing mice was 66.0 ± 14.0% ID/g and 85.6 ± 30.7% ID/g, respectively (Figure 5C,D).

For a more precise determination of the potential of [^68^Ga]KISS1-54, we calculated the tumor-to-muscle and tumor-to-liver ratios for both LNCap and MDA-MB-231 tumors. The tumor-to-muscle ratio for LNCap was 2.0 ± 0.3, and for MBA-MB-231, it was 1.4 ± 0.4. These findings are in line with the visual observations. The LNCap tumors could be delineated from the surrounding tissue in all mice examined. In contrast, in MDA-MB-231 tumors (except for one out of ten), no clear tumor detection was possible. 

In addition, the specificity of the [^68^Ga]KISS1-54 binding for the targeted KISS1 receptor in vivo was determined following the co-injection of [^68^Ga]KISS1-54 with the nonradioactive KISS1-54 at a 100-fold concentration in LNCap-bearing mice. The initial uptake of [^68^Ga]KISS1-54 into tumor xenografts could be blocked by 26% to 74% (Figure 5E).

## 3. Discussion

Kisspeptin is a known metastasis suppressor that triggers wide signal transduction downstream after binding to its G protein-coupled receptor KISS1R, leading to a suppression of metastasis in most cancer types [1,2]. Hence, the loss of KP/KISS1R in tumors has been associated with a poor prognosis for patients [3,6,12,13,14,15,16]. In breast cancer, however, KP/KISS1R has been found to play a diametric role. There is a lot of evidence that the KP/KISS1R system positively correlates with tumor invasiveness and poor prognosis in most breast cancers [24,25]. This discrepancy is not fully understood yet, but, in both cases, the KP/KISS1R system appears to be a reliable tumor-dependent prognostic marker which could be used to predict the progression of the disease and to identify possible therapeutic options [9,12,14,15,21,22], making KISS1R a promising molecular target for molecular imaging and targeted therapy for KISS1R-expressing tumors.

In the present study, we developed [^68^Ga]KISS1-54 as a potential radiotracer for addressing the KISS1 receptor in tumors, starting from the KISS1R selective peptide NODAGA-KISS1-54, which was used as a precursor. The development includes the radiolabeling of the NODAGA-KISS1-54 peptide by the positron emitter Gallium-68 (^68^Ga) and a preclinical evaluation of [^68^Ga]KISS1-54 in cell culture and tumor-bearing mice to assess its suitability as a PET radiotracer for molecular imaging. After the establishment of [^68^Ga]KISS1-54 as a radiotracer for PET diagnostics, an extended application for tumor therapy after a successful radiolabeling of KISS1-54 with α/β^−^-emitting radionuclides is envisaged.

Radiolabeling, purification, formulation, and quality control of the obtained [^68^Ga]KISS1-54 were completed after 35 min, which is an acceptable time for further preclinical investigations and for potential clinical studies [26]. In vitro and ex vivo investigations of the stability showed that [^68^Ga]KISS1-54 is stable in the injectable solutions, as well as in human serum, up to 3 h after preparation.

We first determined the expression of KISS1R, the target of the newly developed [^68^Ga]KISS1-54 radiotracer in selected human tumor cell lines of different tumor types. KISS1R expression was assessed via both immunofluorescence and Western blot. However, the Western blot results revealed that the expression level of KISS1R protein appears to be different in the cell lines. Our findings regarding MCF-7 and MDA-MB-231 cell lines are in line with the results reported by Ziegler et al. and Goertzen et al. [20,27]. Furthermore, Ikeguchi and coworkers reported increased KISS1R expression in hepatocellular carcinoma tissues compared to normal liver tissues. The detected KISS1R expression in HepG2 is in line with these results, as well [14]. SK-BR-3 was selected, assuming low KISS1R expression, as described by Blake et al. Again, our results confirmed the lower level of KISS1R in SK-BR-3 compared to the MCF-7, MDA-MB-231, or HeLa cells [28].

The determined in vitro uptake of about 0.6–4.4% of [^68^Ga]KISS1-54 is comparable to the uptake levels of other radiotracers targeting cell receptors, e.g., RGD- or NRG-peptides that bind to integrin or aminopeptidase N, respectively, expressed on activated endothelial and tumor cells [29,30]. In contrast, the cell uptake of radiotracers that are internalized and trapped (e.g., FDG) is significantly higher. 

The highest cellular accumulation of [^68^Ga]KISS1-54 was measured in LNCap cells, followed by HepG2 and MCF-7. The remaining cells examined showed lower tracer uptake values. Hence, the results do not correlate with the KISS1R protein expression levels in the cells detected by Western blot. Because MCF-7, HeLa, and MDA-MB-231 had the highest KISS1R expression, it was expected that these cells would also have the highest [^68^Ga]KISS1-54 uptake. However, Goertzen et al. previously described a predominant intracellular KISS1R expression in MDA-MB-231 [20]. This could be an explanation for the lower binding of [^68^Ga]KISS1-54 to the KISS1R target and for the relatively low uptake of [^68^Ga]KISS1-54 in MDA-MB-231 cells in vitro. Furthermore, in many cases, tumor cells express KP, so autocrine KP-KISS1R binding could lead to partial receptor blocking, resulting in reduced radiotracer-KISS1R binding. KP expression on the tumor cells and its correlation with [^68^Ga]KISS1-54 uptake in vitro were not investigated in this study but will be addressed in continuing studies.

We further investigated the specificity of the uptake of [^68^Ga]KISS1-54 into the different cell lines via co-incubation with non-radiolabeled KISS1-54 peptide. A significant decrease in radiotracer uptake besides MDA-MB-231 and HCT116 indicated a specific binding of the [^68^Ga]KISS1-54 to KISS1R. These results were corroborated with the results obtained after increasing the KISS1R expression via Decitabine treatment of HCT116. The uptake of [^68^Ga]KISS1-54 could be increased in the HCT116 with higher KISS1R expression (DC treatment), and the elevated uptake could be blocked by co-incubation with KISS1-54.

Finally, we tested [^68^Ga]KISS1-54 in a tumor mouse model. For this purpose LNCap cells with high in vitro [^68^Ga]KISS1-54 uptake and MDA-MB-231 with low in vitro [^68^Ga]KISS1-54 accumulation were selected, and cells were injected subcutaneously into nude mice. [^68^Ga]KISS1-54 accumulated in the LNCap tumor xenografts, whereas no visible tracer accumulation was found in subcutaneously implanted MDA-MB-231 tumor. Accordingly, the calculated LNCap/background ratios were higher in LNCap than in MDA-MB-231. The highest radiotracer uptake in the kidneys and bladder indicated predominant renal tracer excretion. In addition, accumulation was also detected in the liver. Kidney and liver accumulations were comparable to those demonstrated by Dotterweich et al. following an intravenous injection of the KISS1-54 peptide conjugated with a fluorescent dye in mice [31]. 

Despite the positive and promising results in vivo, the total amount of [^68^Ga]KISS1-54 in the tumor tissue remained relatively low. Inhibition experiments injecting nonradioactive KISS1-54 reduced the tracer uptake by only 26 ± 5% in vivo, indicating that [^68^Ga]KISS1-54 is also bound nonspecifically in the tumor. However, it must be taken into account that, due to the limited injection volume, only a 100-fold excess of “cold” KISS1-54 peptide could be injected into the mice. In comparison, the nonradioactive KISS1-54 was co-incubated in the in vitro experiments in 1000-fold excess. 

Nash et al. found that KISS1 could suppress metastasis in the absence of KISS1R, leading to the hypothesis that an unidentified receptor for KISS1 binding might exist [32]. Similar assumptions were made in the context of KISS1 signaling in the central nervous system [33]. If another yet unidentified receptor with lower binding affinity for KISS1 exists, this could explain the relatively low blocking rate after the co-injection of [^68^Ga]KISS1-54 and the “cold” KISS1-54 peptide used as a blocking agent.

In addition, we observed a rapid tracer clearance in the first few minutes. Thus, further [^68^Ga]KISS1-54 circulation was highly reduced, resulting in the relatively low absolute tracer accumulation in the KISS1R-positive LNCap tumor. 

Another aspect which could potentially affect the uptake of [^68^Ga]KISS1-54 in tumors could be its structure. [^68^Ga]KISS1-54 is based on the 54 amino acids of the KISS1-54 peptide, but it has been N-terminally extended by the NODAGA chelator for complexation with ^68^Ga^3+^, which theoretically could reduce the affinity of the radioligand to KISS1R. However, only the first 10 C-terminal amino acids are required for receptor binding [1,2]. For this reason, we assume that the NODAGA-extension does not affect the binding affinity of the ligand. 

In summary, further studies are needed to develop a more optimized KISS1 radiotracer with a higher tumor uptake and tumor-to-background ratio for clinical applications. Nevertheless, to our knowledge, we radiolabeled, for the first time, a KISS1R radioligand for PET-imaging of KISS1R-expressing tumors and successfully evaluated it in cell culture and an in vivo experiment in a tumor xenograft. The initial promising results encourage the further optimization of KISS1R radioligands.

## 4. Material and Methods

### 4.1. Materials

The NODAGA-KISS1-54 peptide used as the starting material for radiolabeling was synthesized commercially by Genaxxon Bioscience (Ulm, Germany) via N-terminal conjugation of KISS1-54 with the NODAGA-chelator. The C-terminal amidated peptide comprises the peptide sequence 68–121 of the full-length peptide encoded by the *Kiss1* gene sequence. The conjugated NODAGA-KISS1-54 precursor was characterized via mass spectrometry, and the chemical purity was proved by HPLC (>99% chemical purity). The mass spectrum and HPLC chromatogram of the compound are provided in the Supplementary Materials.

### 4.2. Radiosynthesis of [^68^Ga]KISS1-54 

[^68^Ga]Ga-NODAGA-KISS1-54 ([^68^Ga]KISS1-54) was synthesized according to a method described previously [34]. Briefly, the synthesis was performed on a synthesis module from Scintomics (Fürstenfeldbruck, Germany). ^68^GaCl_3_ for radiolabeling was eluted with 0.1 M HCl from a ^68^Ge/^68^Ga-generator (GalliaPharm, Eckert & Ziegler, Berlin, Germany) into a reaction vial containing 150 µL of 2.5 M HEPES-buffer and 10 µg of KISS1-54 peptide. After a 10 min reaction time at 90 °C, the product was purified with a Sep-Pak-C18 light cartridge (Waters, Eschborn, Germany), diluted with 0.9% NaCl (B.Braun, Melsungen, Germany), and finally passed through a 0.2 µm sterile filter (Millex-GV, Merck-Millipore, Darmstadt, Germany). The 2-[^18^F]fluoro-2-deoxyglucose ([^18^F]FDG) was prepared in-house at the Interdisciplinary PET-Centre of the University Hospital Würzburg, as described previously [35].

The radiochemical purity of [^68^Ga]KISS1-54 was determined via high-performance liquid chromatography (HPLC). A linear gradient started from 100% H_2_O (0.1% TFA) to 100% acetonitrile (0.1% TFA) over 10 min, with a flow rate of 0.7 mL/min, using a Nucleosil column (100-5 C18 125 × 4.6 mm) (CS-Chromatographie, Langerwehe, Germany) and HPLC system (Knauer, Berlin, Germany). For the determination of the in vitro stability, the [^68^Ga]KISS1-54 solution was diluted in phosphate-buffered saline (PBS) and stored at room temperature, and the HPLC runs were repeated 1 h and 3 h after the end of synthesis.

### 4.3. In Vivo Stability

For the determination of the in vivo stability, [^68^Ga]KISS1-54 was incubated in fresh human serum at 37 °C for 3 h. After 0.5 h, 1 h, 2 h, and 3 h, adequate samples were taken, purified, and analyzed via HPLC under the conditions described above. As serum dilution and purification resulted in a low signal-to-noise ratio in the recorded HPLC radioactivity channel, fractions were collected every 30 s, and radioactivity was measured with a gamma counter (Wizard 2480, Perkin-Elmer, Rodgau, Germany). The measured radioactivity (counts per minute) for each fraction was plotted versus time in a graph. 

### 4.4. Cell Lines and Cell Culture

The human breast cancer cell lines MCF-7, SK-BR-3 and MBA-MB-231 (triple-negative), LNCap (human prostate carcinoma lymph node metastasis), HeLa (cervix carcinoma), HepG2 (hepatocellular carcinoma), and HCT116 (human colon carcinoma) were purchased commercially from DSMZ (Braunschweig, Germany). If not otherwise stated, all cell culture media and supplements were obtained from Life Technologies (Darmstadt, Germany). 

Cells were cultured as described previously [36]. In detail, MCF-7 in RPMI medium containing 10% fetal bovine serum (FBS), 1 mM sodium pyruvate, and 0.1 M nonessential amino acids (NEAA); HeLa and HepG2 in RPMI medium containing 10% FBS and 2 mM Glutamax; MDA-MB-231 in Leibovitz’s L15 with 10% FBS; HCT116 and SK-BR-3 in McCoy’s 5A medium containing 10% FBS and 2 mM Glutamax; and LNCap in RPMI medium containing 10% FBS, 1 mM sodium pyruvate, 0.1 M NEAA, 2 mM Glutamax, and 0.01 M HEPES. All cell culture media were used without antibiotics. MCF-7, HCT116, SK-BR-3, LNCap, HeLa, and HepG2 were maintained at 37 °C in a humidified incubator with a 5% CO_2_ atmosphere and MDA-MB-231 cells were handled without gaseous exchange. The absence of mycoplasma in the cell culture was regularly tested using the Venor GeM qOneStep-Kit (Minerva-biolabs, Berlin, Germany).

### 4.5. Immunofluorescence

Cells were seeded in 24-well plates on microscope cover glasses (VWR, Ismaning, Germany). After fixation/permeabilization, cells were incubated overnight with anti-KISS1R antibody (abcam, Berlin, Germany) or without primary antibody (negative control) and then stained with Alexa-Fluor^®^-488 conjugated secondary antibody (abcam, Berlin, Germany). The nucleus was counterstained with 4′,6-Diamidin-2-phenylindol (DAPI) (Sigma-Aldrich, Deisenhofen, Germany). Fluorescence images were taken on an Axio Scope.A1 fluorescence microscope equipped with a camera and the ZEN2 (blue edition)-Software, version 2.0.0.0 (Zeiss, Jena, Germany). Fluorescence canals were merged using the software FIJI (Image J, version 1.53t).

### 4.6. Western Blot

After counting, cells were lysed with Lämmli/β-mercaptoethanol buffer (1 × 10^6^ cells/100 µL), followed by heating to 90 °C for 5 min. For Western blot, 100,000 cells, respectively, 10 µL was loaded onto an 8% polyacrylamide gel and subsequently separated via electrophoresis. Proteins were transferred to a nitrocellulose membrane via wet transfer, followed by Ponceau-S staining. Before antibody incubation, the membrane was destained with water and then blocked for 1 h with 5% milk/TRIS-buffered saline/0.1% Tween20. The membrane was incubated with the appropriated primary antibodies, anti-GPR54 polyclonal antibody, rabbit, 1:1000 (ab137483, abcam, Berlin, Germany); and anti-lamin A/C, goat, 1:500 (SC-6215, Santa Cruz, Heidelberg, Germany), respectively, at 4 °C, overnight. The next day, the membrane was washed 3 times with TRIS-buffered saline/0.1% Tween20 and then incubated with the horseradish peroxidase-conjugated secondary antibodies: goat anti-rabbit, 1:5000 (170-6515, Bio-Rad, Feldkirchen, Germany); or donkey anti-goat, 1:10,000 (V805A, Promega, Walldorf, Germany). After 1 h of incubation and 3 times of washing with TRIS-buffered saline/0.1% Tween20, visualization was performed using chemiluminescence reagent (Cytiva, Buckinghamshire, UK). Chemiluminescence was recorded on Amersham Imager 680 (GE Healthcare, Chicago, IL, USA).

### 4.7. In Vitro Cell Uptake

Cells were seeded in 24-well plates, and 1 h before [^68^Ga]KISS1-54 uptake, the cell culture medium was replaced by PBS/5% BSA. Cells were incubated at 37 °C with [^68^Ga]KISS1-54 for 5–90 min or co-incubated for 60 min with [^68^Ga]KISS1-54 and KISS1-54 (Sigma Aldrich, Deisenhofen, Germany). After incubation, cells were washed three times with ice-cold PBS/5% BSA and harvested. Radioactivity was measured with a gamma-counter (Wizard 2480, Perkin-Elmer, Rodgau, Germany), and cells were subsequently counted with Countess 3 (Thermo Fisher Scientific, Waltham, MA, USA). In the case of treatment with 5-Aza-2′-Deoxycytidine (Decitabine (DC)), cells were preincubated for 72 h in cell culture medium containing 5 µM Decitabine (Merck Millipore, Darmstadt, Germany).

For data analysis, the determined accumulation of [^68^Ga]KISS1-54 in vitro was standardized as “uptake/100,000 cells” for a better comparison. For example, the cell number differed between pretreatment with and without Decitabine, which is due to the treatment itself, making standardization necessary. 

### 4.8. Animal Studies

The animal studies were conducted according to the principles and procedures outlined in the Guide for the Care and Use of Laboratory Animals and were in line with the Animal Welfare Act and the directive 2010/63/EU. All mice (*CD1-Foxn1nu*) were purchased from Charles River (Sulzfeld, Germany) and held in the animal facility of the University Hospital of Würzburg. After their arrival, they had at least a week to get used to their new environment in the animal facility. 

For implantation, cells were grown to 70–80% confluence, harvested, and placed in PBS.

Then, 5 × 10^6^ MDA-MB-231-cells in PBS and 5 × 10^6^ LNCap cells in Matrigel Matrix^®^ without phenol-red (Corning Life Sciences B.V., Amsterdam, The Netherlands), respectively, were inoculated subcutaneously into the front flank of eight-week-old mice. After implantation, the animals were examined and monitored daily for tumor size and well-being.

### 4.9. µ-PET Imaging

After the tumor reached a size of ≥0.5 cm in diameter, micro-PET scans were started. 

For tracer injection, and during the micro-PET scans, mice were kept under 1.5% isoflurane anesthesia in 100% oxygen. Body temperature was maintained at a physiological level by using heating pads throughout the whole examination period.

Then, 3.0 ± 0.9 MBq of freshly prepared [^68^Ga]KISS1-54 was injected intravenously (i.v.) into the tail vein. The first animals were scanned over 80 min directly after tracer injection to find out the optimal time point for PET acquisition. An analysis of these dynamic PET scans revealed that the optimal tumor-to-background ratio and, thus, the best delineation of the tumors are achieved 60 min post-injection (p.i.). Therefore, data acquisition for all further mice started 60 min p.i. over 20 min.

Each acquired set of data was sorted with Fourier rebinning (FORE) to a 2D dataset of sinograms, which were reconstructed with the OSEM2D reconstruction algorithm. The software AMIDE Medical Image Data Examiner (Version 1.0.4) was used to quantify the radioactivity uptake in various regions of interest (ROIs). For the semi-quantitative analysis of the PET images, spherical ROIs were defined and used. For quantification, tumor-to-muscle, tumor-to-liver, and tumor-to-lung ratios (maximal tumor uptake/mean organ uptake) were calculated. 

### 4.10. Statistical Analysis

The calculated cell uptake and micro-PET data were decay-corrected and expressed as the mean ± standard deviation (SD) or normalized values ± SD. All statistical evaluations were performed using the OriginPro 2017G software (OriginLab Corporation, Northampton, MA, USA). If applicable, means were compared using the unpaired Student’s *t*-test. The *p*-values < 0.05 were considered statistically significant.

## 5. Conclusions

In conclusion, we developed, for the first time, a ^68^Ga-labeled KISS1-54 derivate, [^68^Ga]KISS1-54, with a marked affinity for the KISS1 receptor. The preclinical evaluation in several tumor cell lines and in tumor-bearing mice demonstrates a specific accumulation of the new radiotracer in KISS1R-expressing tumor cells in vitro and in vivo. However, the tumor uptake does not appear high enough for potential clinical applications. Therefore, further improvements are necessary to ascertain the potential of KISS1-54-based radiopharmaceuticals as candidates for the PET imaging of KISS1R-expressing tumors clinically. 

## Data Availability

Data is contained within the article.

## Supplementary Materials

The following supporting information can be downloaded at: https://www.mdpi.com/article/10.3390/ph17010044/s1.

## Abbreviations

BSA	bovine serum albumin
DAPI	4′,6-diamidin-2-phenylindol
DC	5-Aza-2′-Deoxycytidine (Decitabine)
DMEM	Dulbecco’s Modified Eagle Medium
FBS	fetal bovine serum
GPR54	G protein-coupled receptor
HCl	hydrochloric acid
HEPES	4-(2-hydroxyethyl)piperazine-1-ethanesulfonic acid
HPLC	high-performance liquid chromatography
IF	immunofluorescence
i.v.	intravenous
KISS1R	KISS1 receptor
KISS1-54	KISS1 peptide with 54 amino acids
KP	kisspeptin
NaCl	sodium chloride
NEAA	nonessential amino acids
NODAGA	1,4,7-triazacyclononane-1-glutaric acid-4,7-acetic acid
PBS	phosphate-buffered saline
PET	positron emission tomography
p.i.	post-injection
ROI	region of interest
RPMI	Roswell Park Memorial Institute
SD	standard deviation
TRIS	tris(hydroxymethyl)aminomethane
[^18^F]FDG	2-[^18^F]fluoro-2-deoxyglucose
[^68^Ga]KISS1-54	NODAGA-KISS1-54 peptide radiolabeled with ^68^Ga
^68^GaCl_3_	[^68^Ga]gallium chloride
