# Supplementary material for: Gallium-68-Labeled KISS1-54 Peptide for Mapping KISS1 Receptor via PET: Initial Evaluation in Human Tumor Cell Lines and in Tumor-Bearing Mice
Source: Pharmaceuticals (Basel). 2023 Dec 27;17(1):44. doi: 10.3390/ph17010044 (PMC10821118; doi:10.3390/ph17010044)
Supplement: Supplementary file 1 [file pharmaceuticals-17-00044-s001.zip › pharmaceuticals-2752209-supplementary.pptx]

## Slide 1
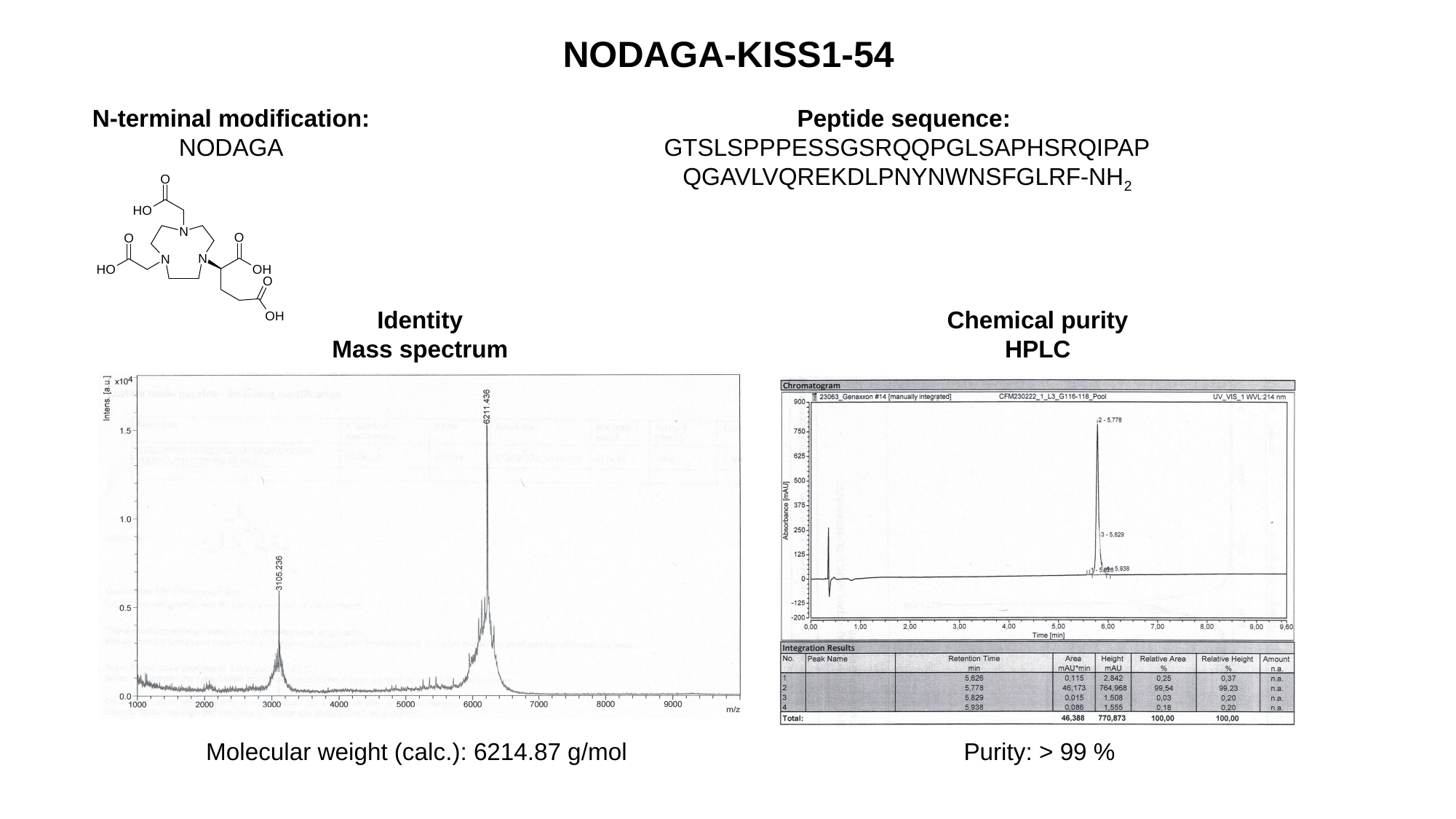

NODAGA-KISS1-54
N-terminal modification: NODAGA
Peptide sequence:
GTSLSPPPESSGSRQQPGLSAPHSRQIPAP
QGAVLVQREKDLPNYNWNSFGLRF-NH2
Identity
Mass spectrum
Chemical purity
HPLC
Molecular weight (calc.): 6214.87 g/mol
Purity: > 99 %
